# ω-3 Long Chain Polyunsaturated Fatty Acids as Sensitizing Agents and Multidrug Resistance Revertants in Cancer Therapy

**DOI:** 10.3390/ijms18122770

**Published:** 2017-12-20

**Authors:** Paola Antonia Corsetto, Irma Colombo, Joanna Kopecka, Angela Maria Rizzo, Chiara Riganti

**Affiliations:** 1 Department of Pharmacological and Biomolecular Sciences, Università degli Studi di Milano, Via D. Trentacoste 2, Milano 20134, Italy; paola.corsetto@unimi.it (P.A.C.); irma.colombo@unimi.it (I.C.); 2 Department of Oncology, Università degli Studi di Torino, Via Santena 5/bis, Torino 10126, Italy; joanna.kopecka@unito.it (J.K.); chiara.riganti@unito.it (C.R.)

**Keywords:** ω-3, PUFA, chemoresistance, membrane, DHA, EPA

## Abstract

Chemotherapy efficacy is strictly limited by the resistance of cancer cells. The ω-3 long chain polyunsaturated fatty acids (ω-3 LCPUFAs) are considered chemosensitizing agents and revertants of multidrug resistance by pleiotropic, but not still well elucidated, mechanisms. Nowadays, it is accepted that alteration in gene expression, modulation of cellular proliferation and differentiation, induction of apoptosis, generation of reactive oxygen species, and lipid peroxidation are involved in ω-3 LCPUFA chemosensitizing effects. A crucial mechanism in the control of cell drug uptake and efflux is related to ω-3 LCPUFA influence on membrane lipid composition. The incorporation of docosahexaenoic acid in the lipid rafts produces significant changes in their physical-chemical properties affecting content and functions of transmembrane proteins, such as growth factors, receptors and ATP-binding cassette transporters. Of note, ω-3 LCPUFAs often alter the lipid compositions more in chemoresistant cells than in chemosensitive cells, suggesting a potential adjuvant role in the treatment of drug resistant cancers.

## 1. Introduction

Several epidemiological studies have highlighted an association between long chain ω-3 polyunsaturated fatty acids (ω-3 LCPUFAs) and incidence of cancers of the breast [1], colon [2], prostate [3], liver [4], and pancreas [5]. Moreover, the ω-3 PUFAs have been shown to improve the efficacy of chemotherapy and radiation against cancer. For example, the efficacy of doxorubicin [6], epirubicin [7], 5-fluorouracil [8], mitomycin C [9], arabinosylcytosine [10], tamoxifen [11], and irinotecan/CPT-11 [12] and of radiation therapy [13] has been shown to be enhanced by ω-3 PUFA association.

Recently, the results of randomized controlled clinical trials supplementing cancer patients with eicosapentaenoic acid (EPA) and docosahexaenoic acid (DHA) during chemotherapy and radiotherapy have been summarized by de Aguiar Pastore Silva et al. [14]. Overall, those studies demonstrated that patients benefit from the combination of ω-3 LCPUFAs and chemotherapy; for instance, fish oils induce weight maintenance or gain and immunomodulation; these achievements reduce inflammation, even when associated with cellular immunosuppression caused by radiotherapy and chemotherapy.

The success of chemotherapy always depends on intrinsic or acquired drug resistance of cancer cells. Tumors are able to tune drug uptake and efflux, acquire qualitative and quantitative alterations of the drug target, increase the efficiency of DNA damage repair systems, and enhance cell death evasion mechanisms, contributing to drug resistance with pleiotropic mechanisms [15].

This review will summarize the evidences sustaining the role of ω-3 LCPUFAs as agents inducing chemosensitization and/or reversing multidrug chemoresistance. The key mechanisms proposed for these effects, with particular attention paid to the impact of ω-3 LCPUFAs on membrane architecture and consequences on drug uptake/efflux and transporter activity, will be discussed.

## 2. In Vitro Evidences of ω-3 LCPUFA’s Positive Effects on Chemosensitization

In vivo and in vitro studies indicate that ω-3 LCPUFAs enhance the sensitivity of cancer cells to chemotherapy [10,16,17,18].

As early as 1979, Burns et al. [19] observed that in L1210 leukemia cells, ω-3 LCPUFA changed plasma membrane fatty acid composition and affected methotrexate transport. The cells, isolated from animals fed with ω-3 LCPUFA-enriched oil, had a lower kinetic constant for methotrexate transport than those isolated from animals fed with a saturated-enriched oil.

Later, Guffy et al. [20] demonstrated that L1210 leukemia cells, grown in medium with DHA, were more sensitive to the adriamycin (ADR) cytotoxicity than cells grown in medium with oleic acid or without fatty acids. In the same period, Ziljlstra et al. [21] showed that also in GLC4 cell line, derived from human small-cell lung carcinoma, the intracellular level of ADR increased when the cancer cells are cultured in DHA-supplemented medium. Furthermore, the drug uptake increased in ADR-resistant cells to a level equal to that of sensitive GLC4 cells. The increased ADR content and cytotoxicity were attributed to the higher levels of DHA and to the significant modifications in plasma membrane phospholipid (PL) composition. In particular, Guffy et al. [20] sustained that, when cell plasma membrane PLs are enriched with ω-3 LCPUFAs, cells become more sensible to lipid peroxidation and more susceptible to membrane damage.

On the same line, Ikushima et al. [22] and Das et al. [23] demonstrated that DHA is also able to increase vincristine cytotoxicity and its uptake in neuroblastoma and cervical cell lines.

In addition, Sturlan et al. [24] showed that DHA strongly increases arsenic trioxide (As_2_O_3_)-mediated apoptosis in the acute myeloid leukemia. As_2_O_3_ has been used for the treatment of acute promyelocytic leukemia (APL) HL60 cells. Different mechanisms have been suggested to explain the anti-leukemic activity of As_2_O_3_, but the generation and accumulation of reactive oxygen species (ROS) is likely the most involved one. Indeed, ROS intracellular accumulation determined the disruption of the mitochondrial membrane potential with the release of cytochrome c, activation of caspases’ cascade, and apoptosis. In particular, Sturlan’s research indicated that the co-treatment of HL60 cells with As_2_O_3_ and DHA increases amounts of ROS and thiobarbituric acid reactive substances (TBARSs), reduces the mitochondrial potential, and activates caspase-3 inducing apoptosis. TBARSs are one of the most widely used markers of lipid peroxidation and are generated in the presence of malondialdehyde (MDA), a reactive aldehyde produced by lipid peroxidation of polyunsaturated fatty acids.

As_2_O_3_ was also used to treat solid cancers, such as neuroblastoma, head and neck cancer, gastric, prostate, and renal carcinoma. In solid tumors, the production and accumulation of ROS appear to be the key mechanism responsible for As_2_O_3_ cytotoxic action [25]. Nevertheless, clinically achievable dosages of As_2_O_3_ are not effective in all tumor types; for this reason, several in vitro studies have been aimed to enhance As_2_O_3_ cytotoxic effects without increasing its concentration. In several preclinical studies, exogenous PUFAs sensitized tumors cells to ROS-inducing anticancer drugs, such as As_2_O_3._ Baumgartner et al. [26] tested breast (MDA-MB-468, SKBR-3, MCF7), cervical (HeLa), ovarian (SKOV-3, ES-2), colon (HT29, SW-620, LS-174T), prostate (PC-3), and pancreatic (PANC1) cancer cell lines that were resistant to As_2_O_3_ or DHA as single agents. Interestingly, their combination reduced viability in SKBR-3, HT29, SW-620, LS-174T, SKOV-3, and PC-3 cells and significantly increased TBARSs.

In 2005, Menendez et al. [27] demonstrated that ω-3 LCPUFAs enhance chemosensitivity by their increasing peroxidative processes and regulating oncoprotein expression. γ-linolenic acid (GLA) resulted in the most potent PUFA increasing paclitaxel toxicity followed by α-linolenic acid, EPA, and DHA, while linoleic acid (LA) had no effects. The authors sustained that there was a strong synergistic cytotoxic effect of DHA and paclitaxel or docetaxel in MDA-MB-231 cells. Moreover, the exposure of BT-474 or SK-BR3 cells to DHA for 24 h reduced p185Her/neu oncoprotein expression up to 78% in BT-474 and to 38% in SK-BR3 cells compared to untreated cells.

The co-treatment DHA and cisplatin was used on human small lung carcinoma GLC4 cell line and its cisplatin-resistant counterpart by Timme-Bosscha et al. [28]. DHA caused a three-fold decrease of cisplatin resistance in cells refractory to the drug. This work suggested that DHA incorporation in cell membrane PLs could increase intracellular cisplatin content, enhancing DNA damage induced by the inter-strand cross-links and nucleotide adducts produced by cisplatin.

## 3. Mechanisms Proposed for Chemosensitizing Effects of ω-3 LCPUFAs

**Figure 1:**
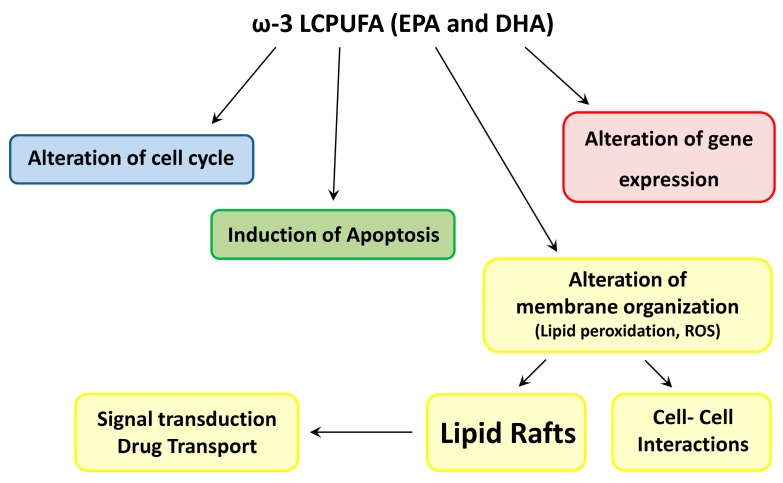
Proposed mechanisms for ω-3 LCPUFA anticancer effects. LCPUFA—long chain polyunsaturated fatty acids; EPA—eicosapentaenoic acid; DHA—docosahexaenoic acid.

The specific mechanisms involved in ω-3 LCPUFA chemosensitizing effects are not fully understood, but nowadays, alteration in gene expression [29], modulation of cellular proliferation [30] and differentiation [31], induction of apoptosis [32], increase in drug transport across the cell membrane, generation of reactive oxygen species (ROS), and lipid peroxidation have been reported (Figure 1). For example, lipid peroxidation is one of the major mechanisms involved in doxorubicin cytotoxicity, together with topoisomerase II inhibition and ROS production [33]. Anthracycline cardiotoxicity represents a major problem for chemotherapy, underlying that the major mechanism is related to drug interference on the respiratory chain and consequent oxidative stress [34]. Since DHA, which has six double bonds, is easily subjected to peroxidation, the increase in the membrane unsaturation index produced by DHA incorporation enhances the ROS content generated from doxorubicin metabolism [35]. This hypothesis is sustained by in vivo and in vitro studies that highlight the correlation between DHA supplementation and increased oxidative stress due to higher lipid peroxidation.

In this context, Vibet et al. [36] demonstrated that the sensitization of breast cancer MDA-MB-231 cells to doxorubicin by DHA is related to a marked decrease in glutathione peroxidase (GPx), the major antioxidant enzyme that uses glutathione as a reductive agent. In particular, the decrease in GPx1 activity in MDA-MB-231 cells was associated with a decreased protein level but not with a decreased mRNA, suggesting a post-transcriptional DHA effect. GPx might be damaged by lipid peroxidation products generated in breast cancer cells treated with DHA and doxorubicin. These oxidant products might reduce GPx activity, probably modifying the selenocysteine residue of the enzyme active site [37]. As a consequence, the inactivated enzyme is degraded by proteases. In the same study, GPx1 activity decreased also in rat tumors after supplementation with EPA/DHA or DHA alone, and this reduction was again associated to an increase of chemosensitivity to anthracyclines.

**Figure 2:**
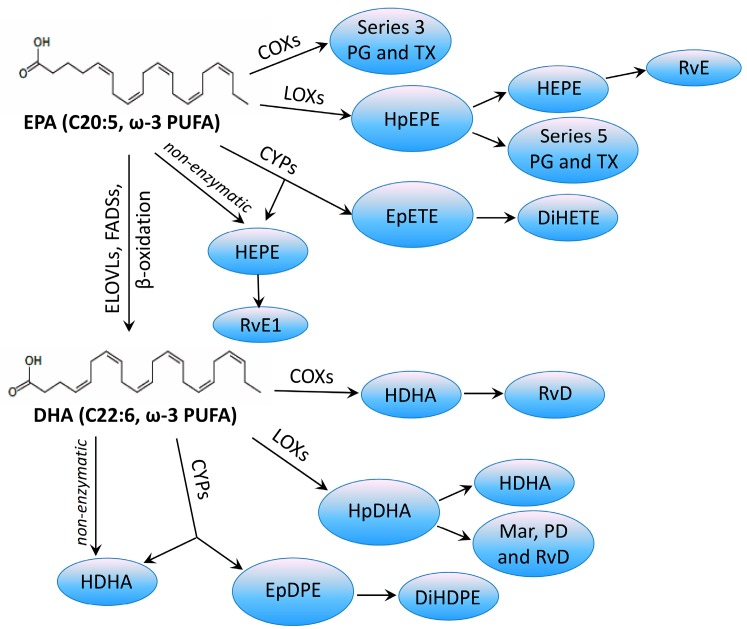
Overview of the key COX, LOX, and CYP-derived metabolites from EPA and DHA. COX—cyclooxygenases; LOX—lipoxygenases; CYP—cytochrome P450; PG—prostaglandin; Tx—thromboxane; HpETE—hydroperoxy eicosatetraenoic acid; HpEPE—hydroperoxy eicosapentaenoic acid; EpETE—epoxy eicosatetraenoic acid; DiHETE—dihydroxy eicosatetraenoic acid; HEPE—hydroxy eicosapentaenoic acid; HpDHA—hydroperoxy docosahexaenoic acid; HDHA—hydroxy docosahexaenoic acid; EpDPE—epoxy docosapentaenoic acid; DiHDPE—dihydroxy docosapentaenoic acid; Lx—lipoxin; LT—leukotriene; Mar—maresin, PD—protectin; RvD—D series resolvins; RvE—E series resolvins.

One of the most important biological functions of ω-3 LCPUFAs is their role as precursors of bioactive lipid mediators, such as eicosanoids and docosanoids (Figure 2). ω-3 LCPUFAs and their metabolites act as second messengers when inserted in the cell membrane. Following the binding of growth factors and hormones to membrane receptors, phospholipase A_2_ (PLA_2_) is activated and releases dihomo-**γ**-linolenic acid (DGLA, C20:3, ω-6), arachidonic acid (AA, C20:4, ω-6), EPA (C20:5, ω-3) and DHA (C22:6, ω-3) from the sn-2 position of PLs. These fatty acids are optimal substrates for eicosanoid biosynthesis mediated by cyclooxygenase (COX), lipoxygenase (LOX) or cytochrome P450 monoxygenase (CYP). High levels of ω-6-derived prostaglandins (PGs) and/or high levels of COX2 have been reported in many human cancers, including those of the breast, cervix, lung, skin, colon, and prostate [38,39], and COX2 inhibitors, such as celecoxib, indomethacin, aspirin, and piroxicam, have been used in experiments aimed to reduce carcinogenesis. The combination of these drugs at low concentrations with proper dietary supplements has been suggested to improve the antitumor effects and decrease the side effects. Negi et al. [40] showed that the combination of celecoxib and ω-3 LCPUFAs is more effective in the treatment of experimentally induced mammary cancers: this effect can be attributed to modifications in redox signaling, changes in c-myc, and p53 expression, apoptosis, and proliferation. In addition, Reddy et al. [41] showed that low doses of celecoxib, associated with a diet containing 10% mixed lipids and 10% fish oil, significantly reduced COX2 activity and expression, and colon cancer incidence compared with low doses of celecoxib associated with a Western-style diet, including saturated fats of animal origin as well as ω-6 LCPUFAs.

The inhibition of carcinogenesis induced by ω-3 LCPUFAs is also mediated through the activation of retinoid X receptors (RXR) and peroxisome proliferator activated receptor (PPAR) [42]. Indeed, Narayanan et al. [43] suggested that a combination of DHA and celecoxib inhibits the carcinogenesis process in three prostate cancer cell lines (LNCaP, DU145, and PC-3) through multiple pathways that involve PPARγ, RXRα, and nuclear factor κB (NF-kB) activity. NF-kB is an inducible transcription factor responsible of regulation of several inflammation- and cancer-related gene expression. The co-treatment with DHA and celecoxib significantly reduces the nuclear translocation of NF-κB-p65 component and blocks p65-induced transcription of genes related to cancer progression. Moreover, ω-3 LCPUFAs inhibit the cleavage of inactive to active sterol response element binding protein 1c (SREBP-1c): this event reduces the synthesis of fatty acids that are the main energy source in prostate cancer, where androgens upregulate fatty acid synthase enzyme (FASN) [44,45]. SREBP-1c is a positive regulator of FASN expression through binding elements in the FASN promoter [46]. The inhibition of SREBP-1c causes the intracellular accumulation of cholesteryl esters and thus promotes cell cycle arrest [47].

Emerging data also sustain the key role of the gut microbiome in mediating the ω-3 LCPUFA beneficial effects on immune and inflammation processes that increase the chemo response. The gut microbiome regulates drug metabolism and functionality and is modified in cancer patients [48]. In murine models, ω-3 LCPUFA incorporation in enterocyte membrane PLs determines changes in microbiome composition and exerts protective effects [49]. Indeed, alterations in the host microbiome might change chemotherapy sensitivity and improve pharmacological success. Unfortunately, the data on humans are still limited, and further studies are needed to demonstrate the complex and potential interplay between ω-3 LCPUFAs and gut microbiome [50].

It is noteworthy that PUFA incorporation greatly affects cell membrane fluidity and structure, especially in membrane microdomains or lipid rafts. The plasma membrane regulates many cell biology aspects, such as morphogenesis, proliferation, migration, differentiation, secretion, and apoptosis. Several studies indicate that ω-3 LCPUFA incorporation in the membrane bilayer may determine dramatic changes in physical–chemical properties, significantly lowering cholesterol solubility [51] and changing the activity of transmembrane proteins, such as growth factors, transporters, and G-protein coupled membrane receptors [52].

## 4. ω-3 LCPUFA Impact on Cell Membrane Function and Lipid Raft Organization

Recently, specific membrane proteins whose activity is modulated by changes in membrane environment emerged as potential therapeutic targets in cancer treatment. This novel approach, defined “membrane lipid therapy” [53], is based on the hypothesis that the use of specific lipids might alter cancer membrane composition and structure, dismantling lipid raft architecture and altering the localization and activity of membrane-associated proteins and down-stream pathways that are crucial for tumor cell growth (Figure 1). The chemical–physical properties of cellular membrane not only influence protein functions but also modify the recruitment and activity of peripheral, amphitropic membrane proteins that interact with membrane lipids [54]. Membrane lipids interact with hydrophobic moieties and residues of membrane proteins by lipid–lipid and lipid–protein interactions. For instance, the ATP Binding Cassette (ABC) transporter activity is closely related to membrane lipid environment: changes in PL and cholesterol content, as well as changes in PL fatty acid composition, might modify the membrane surface properties [55].

Indeed, biological membranes represent two-dimensional solutions where lipids are packed with transmembrane proteins [56] and interact with extrinsic membrane proteins. The main membrane lipid components, including sterols (especially cholesterol), sphingolipids (in particular, sphingomyelin—SM), PLs, such as phosphatidylethanolamine (PE), phosphatidylinositol (PI), phosphatidylserine (PS), and phosphatidylcholine (PC), contribute to the stabilization of membrane architecture.

Cancer cells are characterized by an intense lipid biosynthesis that supports the building of new membranes that work to sustain neoplastic proliferation [57]. According to the existing literature, membrane fatty acid and PL profiles of breast cancer are different compared to normal tissues: breast tumors are characterized by a striking increase in membrane PC and PE, coupled with an increase in PL-induced cell signaling. In addition, an increase of saturated fatty acid-containing PC (16:0/16:0) was correlated with poorer overall survival [58]. Saturated fatty acids make the cell membrane less fluid and their higher content was associated to tumor aggressiveness and chemoresistance [59,60]. The fatty acid length and unsaturation degree might modulate membrane fluidity, phase behavior, permeability, membrane fusion, lateral pressure, and flip-flop dynamics, perturbing the protein–lipid interactions in plasma membranes. The increased unsaturation of PL acyl chains improves their flexibility due to rapid isomerisation [61]; moreover, their fatty acid acyl chain composition modulates the activity of growth and survival signaling pathways.

Cancer cells might acquire fatty acids, not only through de novo synthesis, but also through uptake of exogenous fatty acids obtained by diet or released by cancer-associated adipocytes. Exogenous fatty acids may alter membrane organization if they are integrated in the membrane either as free fatty acids (FFAs) or as constituents of PLs.

Our researches have demonstrated that ω-3 LCPUFA incorporation into cell membrane PLs may alter membrane fluidity, modulate cell signaling [51,62], and enhance ROS production and lipid peroxidation [36]. The PUFA-enriched membranes are thinner and have higher fluidity compared to saturated membranes. Moreover, ω-3 and ω-6 PUFAs display significant differences related to the different saturated chain lengths, which are usually longer in ω-6 PUFAs [63,64].

ω-3 LCPUFAs may alter the optimal protein conformation by changing the membrane’s biochemical–biophysical properties. In the last few years, our group has accumulated several evidences showing that ω-3 LCPUFAs, especially DHA, are incorporated in breast cancer membranes with different specificity for each PL moiety: the enrichment is significant especially in PE, PI, and PC [51]. Biochemical and biophysical approaches have confirmed that DHA incorporation causes morpho-dimensional changes in plasma membranes, in particular in detergent-resistant domains or lipid rafts [62,65]. Lipid rafts are dynamic structures characterized by a relative rigidity and reduced fluidity compared with the surrounding plasma membrane, and may rapidly assemble and disassemble, leading to a dynamic segregation of proteins [66]. They are enriched in cholesterol and sphingo- and glycerol-lipids containing saturated fatty acids, and contain several receptors, channels, and transporters, whose localization in raft or non-raft regions modulates their function. In cancer cells, many signaling proteins and receptors regulating pro-oncogenic and apoptotic pathways during the early, advanced, and metastatic stages of tumorigenesis are localized in lipid rafts [67]. Furthermore, lipid rafts and their main component, cholesterol, are quantitatively higher within the membrane of cancer cells than in normal cells [68].

**Figure 3:**
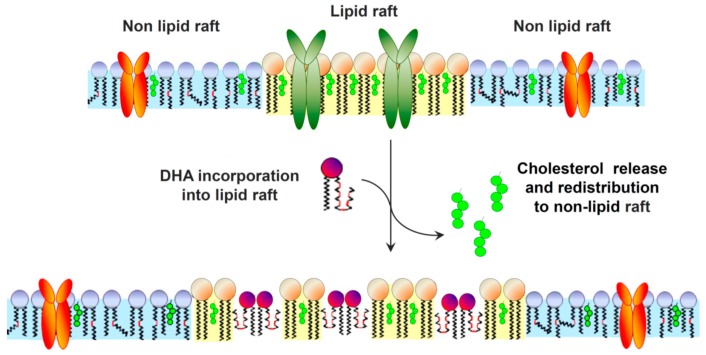
DHA impact on lipid raft structure. DHA incorporation in membrane affects lipid raft organization inducing a shift from cholesterol/saturated fatty acid-rich domains to ω-3 LCPUFA-rich/cholesterol-poor domains, which exhibit different height ranges.

ω-3 LCPUFAs and their metabolites are inserted into lipid rafts with different yields, altering fatty acid composition without decreasing the total percentage of saturated fatty acids typical of rafts. Interestingly, estrogen-resistant breast cancer MDA-MB-231 cells, which display the highest content of cholesterol and saturated fatty acids, had the lowest incorporation of DHA, likely due to sterical hindrance reasons; nevertheless, DHA was able to decrease cholesterol concentration in lipid rafts (Figure 3). Moreover, in two breast cancer cell lines (MCF-7 and MDA-MB-231), the DHA treatment determined a 20–30% decrease in lipid rafts. It is worth noting that, after DHA incorporation, lipid rafts exhibited different height ranges [51]. These alterations influenced the conformation of resident proteins and switched signaling proteins on or off [69].

In conclusion, ω-3 LCPUFAs might dismantle the lipid raft structure and thereby the protein lateral distribution (Figure 3). The poor affinity between ω-3 PUFAs and cholesterol determines a shift of cholesterol out of the raft, inducing de-clustering of membrane microdomains. ω-3 LCPUFA incorporation in membrane microdomains determines a re-localization of raft-associated proteins, e.g., it induces the shift of proteins from rafts into non-rafts or in cytosolic compartment. For instance, ω-3 PUFA acyl chain enrichment in lipid rafts and the subsequent raft de-clustering force the major histocompatibility complex (MHC) class I proteins to shift from rafts into non-rafts compartments [70].

Furthermore, the specificity of PUFA incorporation within PL moieties might influence the synthesis of PUFA-derived mediators (such as prostaglandins, prostacyclins, leukotrienes, resolvines, and protectines) and signal transduction depending on these metabolites.

By altering membrane organization, ω-3 PUFAs, in particular DHA, affect anticancer drug uptake, either by increasing sensitization of cancer cells or by modulating chemoresistance.

## 5. ω-3 LCPUFAs as Revertants of Multidrug Resistance: In Vitro Evidences

Chemoresistance, in particular the simultaneous resistance towards different chemotherapeutic agents known as multidrug resistance (MDR), is one of the most serious problems encountered during chemotherapy. MDR can be present at the diagnosis or can be induced by the selective pressure of chemotherapy and relies on different mechanisms, such as increased drug efflux, reduced drug uptake owing to changes in lipid membrane composition, increased drug sequestration within endo-lysosomes followed by exocytosis, enhanced metabolic inactivation of the drug, and quantitative or qualitative changes in the drug target [71]. The most common event characterizing MDR cells is the overexpression of ABC transporters, such as P-glycoprotein (Pgp), MDR-related proteins (MRPs), and breast cancer resistance protein (BCRP). Together, they efflux classical chemotherapeutic agents (e.g., anthracyclines, taxanes, Vinca alkaloids, epipodophyllotoxins, topotecan, and methotrexate) and targeted-therapies (e.g., imatinib, dasatinib, lapatinib, gefitinib, sorafenib, and erlotinib), limiting their intracellular accumulation and cytotoxicity [72].

Since ω-3 LCPUFAs induce good chemosensitization in drug-sensitive cancer cells, some have started to analyze whether and how they have benefits as MDR-reversing agents. Interestingly, ω-3 LCPUFA effects appeared rather selective for chemoresistant cells because they induced strong chemosensitization in cells with an acquired chemoresistant phenotype compared with the chemosensitive parental clones [21,73] and in non-transformed cells [74]. The chemosensitizing effects, however, were not comparable between different tumors, because different cancer cell lines, even if derived from the same tissue, have different metabolic pathways for ω-3 PUFAs [75]; such variability may explain the discrepancies obtained by using different PUFAs and different cancer cells.

ω-3 LCPUFAs act as MDR reversing tools by pleiotropic mechanisms. Since they are well incorporated in plasma membrane PLs and in particular in lipid rafts, this was one of the first mechanisms investigated. ω-3 LCPUFAs incorporation was correlated with an increased ratio between drug uptake and drug efflux [23]. Interestingly, the changes in lipid compositions induced by ω-3 LCPUFAs are more pronounced in chemoresistant cells than in chemosensitive cells [21], likely as a consequence of the different membrane composition that characterizes these two cell populations [76]. An increased incorporation of saturated fatty acids consequent to the de novo lipogenesis has been associated with an increased resistance to doxorubicin [60]. Opposite effects should be expected when unsaturated fatty acids are incorporated in tumor cell plasma membranes. Indeed, for drugs entering cells by passive diffusion, such as anthracyclines, Vinca alkaloids, and purine analogues, an increased membrane fluidity favored drug uptake [23,77,78]. In Pgp-overexpressing/vincristine-resistant neuroblastoma cells, DHA and GLA increased the intracellular retention of the drug. This event was not due to changes in vincristine efflux, but to the inversion of PUFAs/mono-unsaturated fatty acids (MUFAs) ratio in plasma membrane [23], suggesting that an enhanced uptake, more than a reduced efflux, was responsible for the higher accumulation of vincristine. This conclusion, however, was partially in contrast with the experimental evidences gathered in doxorubicin-resistant breast cancer cells and in vinblastine-resistant nasopharyngeal cancer cells: in both models, DHA reduced the efflux of the Pgp substrate rhodamine 123 [79], leading to the hypothesis that the higher accumulation of doxorubicin and vinblastine detected in DHA-treated cells was due to their reduced efflux.

As noted above, several ABC transporters mediating MDR are highly sensitive to changes in the lipid plasma membrane. Pgp activity for instance is activated by a saturated fatty acid (SFA)-rich environment and is inhibited by increased levels of MUFAs and PUFAs in the plasma membrane [80]. The depletion of SFAs from drug-resistant cell membranes also decreased the amount of Pgp within lipid-rafts [80], where the protein is abundant and active [81,82]. We recently reported that DHA and EPA were highly incorporated in the lipid rafts of colon cancer HT9/MDR cells [73]: following this incorporation, they reduced the amount of total membrane- and lipid raft-associated Pgp and MRP1 (another ABC transporter enriched in rafts [83]), restoring the chemosensitivity to doxorubicin and irinotecan. These MDR-reversing effects were peculiar of ω-3 LCPUFAs, not of the ω-6 arachidonic acid: ω-3 LCPUFAs are indeed highly flexible structures and, compared with ω-6 LCPUFAs, can produce a stronger disassembly of the ordered lipid raft structure. Not all the ABC transporters contained in lipid rafts, however, are inhibited by ω-3 LCPUFAs: lipid raft-associated BCRP, for instance, was increased in DHA-treated cells [73]. BCRP has a less hydrophobic structure than Pgp and MRP1 [71]; in this case, the enrichment of ω-3 LCPUFAs might favor the retention of BCRP in raft compartments instead of promoting its shift in non-raft fractions. According to these data, ω-3 LCPUFAs should be considered able to reverse the resistance towards Pgp and MRP1 substrates. Their efficacy in reversing the resistance towards substrates of other ABC transporters that are not localized in lipid rafts or dependent upon the membrane fluidity has not been clarified. According to the evidences accumulated on MDR cells, ω-3 LCPUFAs are not general ABC transporter inhibitors: their efficacy appears restricted to selected groups of chemotherapeutic drugs and ABC transporters. Since each drug can be effluxed by more than one ABC transporter [71] and MDR cells often express more than one transporter, this consideration freezes the enthusiasm of using ω-3 LCPUFAs as a panacea for MDR tumors.

**Figure 4:**
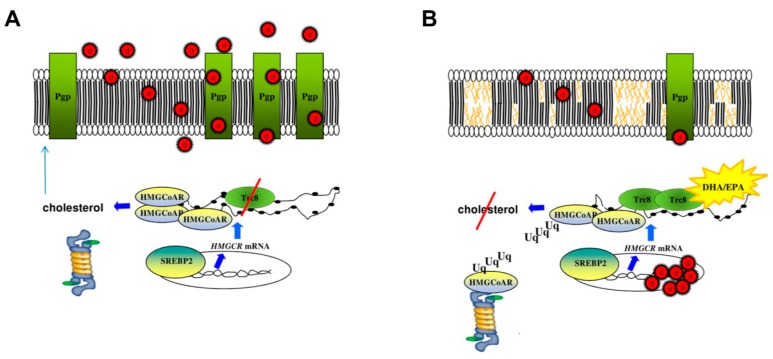
ω-3 LCPUFAs reverse chemoresistance induced by P-glycoprotein by modulating cholesterol synthesis and altering membrane lipid microenvironment. (**A**) MDR cells have a high synthesis of cholesterol, owing to the constitutive over-expression of the enzyme 3-hydroxy-3-methlyglutaryl coenzyme A reductase (HMGCoAR). This is independent of the activation of the transcription factor sterol regulatory binding protein-2 (SREBP2) but is due to the lower activity of the E3-ubiquitin ligase Trc8. A high cholesterol content in the plasma membrane sustains the activity of P-glycoprotein (Pgp), which effluxes several chemotherapeutic drugs (d); (**B**) DHA and EPA are allosteric activators of Trc8 and increase the Trc8-mediated ubiquitination (Uq) of HMGCoAR, reducing cholesterol synthesis. Such cholesterol depletion, together with the incorporation of DHA/EPA in plasma membrane, alters the cholesterol rich/saturated fatty acids rich lipid micro-domains such as lipid rafts, reduces Pgp surface level and activity. As a result, ω-3 LCPUFAs increase the intracellular retention of Pgp substrates, chemosensitizing resistant cells.

Besides SFAs, cholesterol is a second component abundant in lipid rafts. Of note, cholesterol content is higher in the plasma membrane of chemoresistant cells than of chemosensitive cells [73,84]. Pgp activity is strictly dependent on membrane cholesterol: cholesterol depletion induces the shift of Pgp from lipid rafts to non-lipid raft compartment [85,86] and reduces Pgp efflux activity [84]. DHA and EPA, which were well incorporated in lipid rafts, displaced cholesterol from the raft fractions of colon cancer HT29/MDR cells [73]: in agreement with other experimental observations [85,86], the decrease in cholesterol displaced Pgp from lipid rafts and lowered its activity [73]. MDR cells have a higher rate of synthesis of cholesterol and isoprenoids, which increase the expression of Pgp by activating the transcriptional axes Ras/ERK/HIF-1α and RhoA/RhoA kinase/HIF-1α [87]. Interestingly, besides reducing the cholesterol amount in lipid rafts, DHA and EPA also reduced the endogenous synthesis of cholesterol in colon cancer HT29/MDR cells. Indeed, they allosterically activated the E3-ubiquitin ligase Trc8, which promotes the degradation of the cholesterol-pacemaker enzyme 2-hydroxy-3-methylglutaryl coenzyme A reductase (HMGCoAR) [73]. This effect was specific to ω-3 LCPUFAs, because ω-6 AA was ineffective. This discrepancy may be due to the different tridimensional conformation of ω-3 and ω-6 LCPUFAs that makes only the former suitable allosteric activators of Trc8. The reduction of the endogenous cholesterol synthesis further contributed to the depletion of cholesterol from the plasma membrane, reducing the activity of Pgp and overcoming the resistance to the Pgp substrates doxorubicin and irinotecan [73] (Figure 4).

The impact of DHA and EPA on the endogenous cholesterol synthesis, however, is rather controversial [88,89,90] and depends on tumor types and species analyzed, and on the presence or absence of a MDR phenotype. Therefore, notwithstanding the promising results obtained in specific cell lines in vitro, we cannot state *bona fide* that the effects of DHA and EPA as Pgp inhibitors are valid for all tumors.

Few works described LCPUFAs as direct inhibitors and downregulators of Pgp. For instance, ω-3 and ω-6 LCPUFAs decreased the transcription of Pgp in colorectal cancer cells: such a decrease, however, was very small if compared with the marked increased efficacy of paclitaxel, a substrate of Pgp, induced by LCPUFAs in these cells [91], leading to the hypothesis that the changes in Pgp activity or distribution of more than in Pgp expression, are mainly responsible for chemosensitization. A side effect of LCPUFAs in colon cancer cells is the increased expression of the transcription factors constitutive active/androstane receptor CAR and pregnane X receptor PXR [91], which are Pgp inducers [92]. This side effect may attenuate the downregulation of Pgp. On the other hand, LCPUFAs reduce the activity of NF-κB, which also induces Pgp [93], counteracting the CAR- and PXR-mediated increase of Pgp [94]. The complex balance between Pgp transcriptional inducers and repressors is not always easy to unveil, and makes hard to predict a priori whether LCPUFAs work as Pgp downregulators or inducers in a specific tumor.

Although the changes in drug uptake and efflux have been primarily considered responsible for the chemosensitization induced by ω-3 LCPUFAs, other biochemical mechanisms inducing MDR, such as the activity of detoxifying and in activating/inactivating enzyme, can be modulated by ω-3 LCPUFAs. Many chemotherapeutic drugs, including anthracyclines and platinum salts, are metabolized and effluxed as glucuronic acid-, GSH- or sulfate-conjugates via MRP1 [95]. The gene profiling analysis of colon cancer cells pointed out that DHA and EPA downregulated specific isoforms of glucuronyltransferase, glutathione S-transferase (GST), and sulfotransferase, as well as members of the cytochrome P450 family [96]: such reduction of Phase I and Phase II enzymes is expected to reduce the metabolic inactivation of chemotherapeutic drugs and their subsequent efflux, thereby increasing the intracellular accumulation of the active agents/metabolites. A robust expression pattern of antioxidant enzymes is also protective towards the oxidative damages induced by doxorubicin and cisplatin, and is associated with the MDR phenotype [97]. Interestingly, DHA has been reported to reverse doxorubicin resistance in breast cancer MDA-MB-231 cells by decreasing the activity of GPx [36]. This effect, however, cannot be generalized, since GPx was unaffected by DHA in other breast cancer cell lines such as MCF7 cells [36], and it was increased, together with superoxide dismutase, catalase, and GST-π, in non-small cell lung cancer A549 cells [98]. The different rate and pathways of DHA uptake and metabolism, as well as the plethora of different transcriptional factors, co-activators, and co-repressors affecting the expression of antioxidant enzymes, may account for the variable effects of the same ω-3 LCPUFAs in different cell models.

It seems clear that ω-3 LCPUFAs are not general MDR reversing agents, but they may overcome the resistance in a chemotherapeutic agent- and tumor type-dependent way. Their chemosensitizing effects are sometimes also independent from the changes in plasma membrane lipid composition and in drug-metabolizing enzymes. For instance, DHA reversed the resistance to cisplatin in a small cell lung carcinoma cell line by enhancing the formation of DNA inter-strand cross-links [28]. The resistance to gemcitabine in pancreatic cancer is specifically associated with the increased ratio between the pro-survival NF-κB transcription factor and the pro-apoptotic protein poly (ADP-ribose) polymerase (PARP): since ω-3 LCPUFAs inhibited NF-κB and activated PARP, they were particularly suitable in restoring gemcitabine cytotoxicity in this model [99].

The effects of ω-3 LCPUFAs on specific tumor subpopulations and/or on tumor stromal cells may represent additional factors contributing to MDR reversion. Cancer stem cells are the most resistant component of the tumor bulk and are often responsible for tumor relapses. Recently, EPA has been found to increase cell differentiation and deplete cancer stem cells from the colorectal COLO 320 DM cell line: these events restored the efficacy of oxaliplatin and 5-fluorouracil in differentiated and stem cell populations, and chemosensitized stem cells subpopulation to 5-fluorouracil [13]. It has not been investigated whether the chemosensitizing effect in cancer stem cells is shared with other tumor types; in the case of a positive answer, this could represent an added value of ω-3 LCPUFAs as chemosensitizer agents.

Aggressive tumors have often a fast and disordered growth that is not adequately supported by the tumor vasculature: the reduced supply of blood and oxygen limits delivery and activity of chemotherapeutic drugs. By modulating the endothelial synthesis of nitric oxide, ω-3 LCPUFAs increased tumor vasculature and improved the delivery and extravasation of docetaxel in rats bearing drug-resistant mammary tumors [100]. A similar effect was observed in rats with epirubicin resistant mammary tumors treated with DHA [101]: in this model, however, ω-3 LCPUFAs decreased tumor vascularization, suggesting an anti-angiogenic effect of ω-3 LCPUFAs. Since ω-3 LCPUFAs were administered before chemotherapy, two sequential mechanisms may occur in this case: the reduced angiogenesis exerted by ω-3 LCPUFAs can decrease the tumor bulk; such a reduction can make the tumor more eradicable by the subsequent administration of epirubicin. In a neuroblastoma xenograft model, fish oil did not reduce the microvesssel density when administered alone, but it did so when co-administered with sunitinib [102]; in the same condition, fish oil altered the production of local eicosanoids and decreased tumor-associated inflammatory cells, which may include tumor associated macrophages type I. The sum of all these effects produced a significant reduction in tumor growth [102].

Although contrasting in mechanisms, these studies suggest that ω-3 LCPUFAs share the properties of reversing drug resistance in vivo through concurrent mechanisms targeting both either tumor cells or tumor microenvironment.

## 6. ω-3 LCPUFAs as Revertants of Multidrug Resistance: Preclinical and Clinical Studies

Curiously, several studies have demonstrated the chemosensitizing efficacy of LCPUFAs in pre-clinical models before in vitro studies investigated the molecular mechanisms at the basis of such chemosensitization. Despite the differences in the amount, type, and proportion of ω-3 and ω-6 LCPUFAs, all in vivo studies reported that the dietary supplementation with ω-3 LCPUFAs improved the efficacy of chemotherapy in solid and hematologic xenograft tumors [6,10,12,18,102,103] and in spontaneously developing or syngeneic tumors [100,101]. This effect was also valid in advanced tumor stages: for instance, dogs fed with menhaden fish oil and arginine before and after remission of Stage III lymphoblastic lymphoma showed prolonged disease free survival and overall survival compared with animals fed with a standard diet [104].

When given separately and not in a mixture like fish oil, the effects of ω-3 LCPUFAs are, however, more controversial: for instance, whereas DHA alone enhanced the efficacy of epothilone but not the efficacy of 5-fluorouracil and cyclophosphamide, and EPA produced the opposite effect, neither DHA nor EPA increased the antitumor efficacy of gemcitabine in mice bearing colon cancers [105].

Compared with the administration of a single ω-3 LCPUFA, the supplementation with a mixture alters the balance between SFAs and LCPUFAs more deeply within tumor tissues. The changes in the membrane lipid environment, the increase of lipid peroxidation products, and the modifications of eicosanoids produced in tumor stroma—three possible mechanisms of chemosensitization—are more pronounced after ω-3 LCPUFA mixture than a single ω-3 LCPUFA administration. This difference may explain why the dietary supplementation with fish oil improved the efficacy towards many different chemotherapeutic drugs, whereas the supplementation with single ω-3 LCPUFA had doubtful efficacy.

The presence of other nutritional supplements may represent an additional confounding factor: for instance, the supplementation with ω-3 LCPUFAs or glutamine as single agents increased the efficacy of 5-fluorouracil in colon cancer bearing mice, but this event was surprisingly reduced by their simultaneous administration [106]. Moreover, the schedule of ω-3 LCPUFA administration widely varies between each work, making difficult the comparison between preclinical studies. Most protocols gave ω-3 LCPUFAs immediately after the tumor implantation and before chemotherapy and continued with a combined administration of ω-3 LCPUFAs and chemotherapy [6,10,12,18,100,101]. The administration of ω-3 LCPUFAs before or together sunitinib did not produce significant differences in the growth of neuroblastoma xenografts [102], but the comparison between different protocols of ω-3 LCPUFAs and chemotherapy administration has not been performed in other tumor models, leaving several issues uninvestigated. Whether the preventive supplementation of ω-3 LCPUFAs reduces the onset of chemoresistance and whether the administration of ω-3 LCPUFAs at tumor diagnosis or during tumor recurrences are equally effective in overcoming MDR are still unresolved questions. Only when these questions are answered can more precise information about the most effective scheme of ω-3 LCPUFA administration be inferred.

One interesting finding obtained from in vivo studies is that the supplementation with ω-3 LCPUFAs increased the benefits of chemotherapy in both drug-sensitive [103,106,107] and drug-resistant tumors [6,18,101,105,107,108]: in the latter, however, ω-3 LCPUFAs usually produced stronger benefits in terms of tumor regression or stabilization. The higher efficacy of ω-3 LCPUFAs in chemoresistant tumors may be explained by their greater cholesterol synthesis and peculiar membrane lipid composition that affects ABC transporter expression and activity [73].

One pioneering clinical study reported a direct correlation between the positive response to chemotherapy and the high level of ω-3 LCPUFAs, in particular DHA, in adipose breast tissue [109]. This study suggested the possibility that raising the concentration of LCPUFAs might improve chemotherapy efficacy and opened the way to clinical trials investigating the effects of ω-3 LCPUFA supplementation in patients subjected to chemotherapy.

A Phase II trial in patients with metastatic breast cancer showed that the daily supplementation with DHA was well-tolerated in patients receiving anthracycline-based chemotherapy and produced a significant increase in the overall survival, which was directly correlated with blood DHA concentration [110].

Being often refractory to standard chemotherapy, non-small cell lung cancer is a tumor under intensive investigation for the development of new therapeutic approaches. Interestingly, in non-small cell lung cancer patients, first-line chemotherapy supplemented with fish oil improved the clinical response and the overall survival, without increasing the burden of chemotherapy-induced side effects [111]. Since the fish oil supplements used in the study included 2.2 g of EPA and 240–500 mg of DHA, the therapeutic benefit was likely attributable to both LCPUFAs. The adequate intake of ω-3 LCPUFA ranges from 1.1 to 1.6 g/day in adults, with at least 10% DHA and EPA. Although the amount of DHA and EPA given in the above-cited study was higher than the average adequate intake recommended in a standard Western diet, this amount was well also tolerated by debilitated patients, such as patients undergoing chemotherapy treatment. The lack of adverse effects of ω-3 LCPUFAs and/or the attenuation of the chemotherapy side effects [109,111] are added values that make the compliance of patients in the clinical studies relatively high. The amount and types of LCPUFAs necessary to achieve therapeutic benefits in cardiovascular diseases and dyslipidemic syndromes are well known by clinicians. Such experience reduces the difficulty of translating the administration of LCPUFAs to other clinical settings, such as those of oncological diseases. The low cost of ω-3 LCPUFAs [112] compared with the costs of the targeted therapies used in patients unresponsive to conventional chemotherapy is another appealing factor that makes ω-3 LCPUFA supplementation particularly suitable for large population studies.

An alternative to the DHA administration and chemotherapy as single agents is the use of a multitarget conjugate of DHA and chemotherapeutic drug. Two independent trials reported that a DHA-paclitaxel conjugate induced less side effects than free paclitaxel in patients with resistant solid tumors, owing to the different pharmacokinetic profile of paclitaxel released from DHA [113,114]; the conjugate also produced a good stabilization of the tumor in patients refractory to previous chemotherapeutic regimens [114]. The kinetic profile has not been investigated in these studies, and mechanisms of DHA dissociates from paclitaxel: since the two agents are bound by an acyl link [115], it is likely that plasma esterases released DHA from paclitaxel and that the observed tumor stabilization was due to the chemosensitizing effect of DHA on resistant cells. Compared with single agents, multitarget drugs have a lower risk of drug–drug interaction, a better compliance for patients and a more predictable pharmacokinetic profile: given the good chemosensitization efficacy achieved by the DHA-paclitaxel conjugate, this approach may represent a useful tool for future Phase II and Phase III trials.

## 7. Concluding Remarks

Despite a certain variability in the mechanisms, types, doses, and schedule of ω-3 LCPUFA administration, most studies have demonstrated that DHA and EPA improve the efficacy of chemotherapy in vitro and in vivo. It is noteworthy that the chemoresistance is higher, and the chemosensitizing effect is also higher and is a feature that is uncommon for other MDR reversing agents or ABC transporter inhibitors. Moreover, ω-3 LCPUFA supplementation was generally well tolerated and did not increase the side effects of chemotherapy: some studies indeed have reported a reduction of tumor-related or chemotherapy-related side effects, such as cachexia [10], osteoporosis [116], neutropenia [18], cardiotoxicity [117], and diarrhea [12,106]. LCPUFA uptake by tumor and non-tumor cells is highly variable [110,118], leading to rule out that the antitumor effects of LCPUFAs only depend on the higher uptake of LCPUFA in tumor cells. It is well-known that LCPUFAs change the lipid membrane composition of transformed and non-transformed cells in a different way [119], and that the membrane composition of drug-sensitive and drug-resistant cells is different, the latter being richer of cholesterol and lipid rafts [76]. It is likely that the incorporation of ω-3 LCPUFAs, which lower plasma membrane cholesterol and disrupt lipid raft architecture, impairs the activity of membrane proteins more in drug-resistant cells than in drug-sensitive cells or in non-transformed cells. This may explain why the chemosensitizing effects of ω-3 LCPUFAs were often more pronounced in MDR cells.

In addition, ω-3 LCPUFAs target specific metabolic pathways that are necessary for the maintenance of the MDR phenotype [73]. The search for compounds exerting a selective cytotoxicity in MDR cells—an event known as “collateral sensitivity” [120]—is very active. ROS inducers, ATP depleting agents, detergents increasing membrane fluidity are the most promising agents in this new generation of MDR reversing tools [120]. However, the potential toxicity of these compounds in non-transformed cells raises some doubts about their extensive use in vivo. ω-3 LCPUFAs are a step over these “collateral sensitivity” inducers, because they are more effective in MDR cells and well-tolerated in patients.

A considerable number of in vivo studies have shown a good chemosensitizing effect of ω-3 LCPUFAs in tumors resistant to anthracyclines and taxanes [18,100,101,110,113,114], two classes of drugs widely used in both hematological and solid malignancies. This feature enlarges the potential number of oncological patients who may benefit from ω-3 LCPUFA supplementation, compared with other MDR reversing compounds, and makes the realization of Phase III trials easier.

On the other hand, although in vitro and in vivo studies highlighting the therapeutic benefits of ω-3 LCPUFAs have been abundant in the last two decades, several issues must still be clarified, before their extensive use in clinical practice is proposed. First, most attention has been focused on the effects of ω-3 LCPUFAs on the lipid plasma membrane and plasma-membrane-associated proteins. LCPUFAs can be theoretically incorporated in all cell membranes, thus affecting the lipidomic/proteomic profile of the endoplasmic reticulum, the Golgi apparatus, endosome/exosome vesicles, mitochondria, and the nucleus. In all these organelles, transmembrane proteins regulate crucial biological functions; the extent of LCPUFA incorporation in these intracellular membranes and the impact on the physiological organelle activity are a subject largely unexplored. Specific investigations in the field may unveil new mechanisms at the basis of antitumor and MDR reversing efficacy of ω-3 LCPUFAs.

In contrast with most evidence showing that LCPUFAs chemosensitize cancer cells, a recent work reported that cisplatin-treated colon carcinoma became resistant to different chemotherapeutic drugs following the cisplatin-induced production of two endogenous LCPUFAs, namely 12-oxo-5,8,10-heptadecatrienoic acid and hexadeca-4,7,10,13-tetraenoic acid (16:4, ω-3), by mesenchymal stem cells [121]. This work is the only one reporting that endogenous ω-3 LCPUFAs, in contrast with the exogenously administered ones, have a deleterious effect on chemotherapy efficacy, opening a second field of investigation that is actually unexplored.

A third issue poorly known is represented by the inter-individual differences in LCPUFA absorption, by the genetic polymorphisms in the enzymes involved in fatty acid uptake, transport, and metabolism, and by the amount and types of other fatty acids present in the patient diet. In light of these factors, a careful optimization of the ω-3 LCPUFA supplementation protocol, tailored to single patients, might be required. At present, how such inter-individual differences affect ω-3 LCPUFA efficacy is not known; only large population studies will likely clarify these points. The safety and the low cost of ω-3 LCPUFA supplementation may be advantageous in realizing such studies, increasing the confidence that most of the open questions concerning mechanisms and benefits of LCPUFAs as chemosensitizing agents will be solved soon.
